# What constitutes safe and effective dose titration of methadone and buprenorphine/naloxone? protocol for a population-based target trial emulation

**DOI:** 10.64898/2026.07.23.26358700

**Published:** 2026-07-27

**Authors:** Momenul Haque Mondol, Michelle Zanette, Jeong Eun Min, Megan Kurz, Robert William Platt, Shaun Seaman, Paxton Bach, Mohammad Ehsanul Karim, Maria Eugenia Socías, Paul Gustafson, Jason M Sutherland, Bohdan Nosyk

## Abstract

**Introduction:**

Clinical guidelines for managing opioid use disorder (OUD) recommend incremental dose titration for methadone and buprenorphine/naloxone to achieve a safe therapeutic maintenance dose. Dose titration recommendations are based on clinical experience, pharmacologic rationale, and expert consensus, with limited real-world evidence, especially in settings with widespread fentanyl use, where traditional titration schedules may be insufficient to address the higher opioid tolerance among people initiating treatment. This study aims to determine the comparative effectiveness of alternative titration schedules on completed induction and time to all-cause mortality.

**Methods and analysis:**

We will conduct a population-based retrospective cohort study using linked data from nine provincial health administrative databases. The study population will include adults (≥18 years) in British Columbia, Canada, who initiated methadone or buprenorphine/naloxone between 01/01/2010 and 30/06/2022. Primary outcomes are completed induction (defined as no dose increase for ≥two weeks without any intervening decrease) and time to all-cause mortality, with overdose-related acute care visits or overdose-related death and treatment discontinuation as secondary outcomes. Using a target trial framework, this study will implement a ‘clone-censor-weight’ approach to estimate the per-protocol effects of sustained titration schedules capturing adherence to and deviation from clinical guidelines. Sensitivity analyses will assess the robustness of findings through cohort and timeline restrictions as well as alternative exposure and outcome definitions.

**Discussion:**

This study will generate real-world evidence on the comparative effectiveness of alternative titration schedules of methadone and buprenorphine/naloxone on completed induction and all-cause mortality. The findings will support evidence-informed updates to OAT guidelines and clinical decision-making in British Columbia and other jurisdictions facing escalating opioid-related harms.

## Full Text Availability

The license terms selected by the author(s) for this preprint version do not permit archiving in PMC. The full text is available from the preprint server.

